# *In vivo l*ow frequency MR-guided thalamotomy with focused ultrasound: thermal *vs* mechanical lesioning in pig brain

**DOI:** 10.1186/2050-5736-3-S1-P41

**Published:** 2015-06-30

**Authors:** Zhiyuan Xu, Carissa Carlson, John Snell, Matt Eames, Arik Hananel, M Beatriz Lopes, Prashant Raghavan, Cheng-Chia Lee, Chun-Po Yen, David Schlesinger, Neal Kassell, Jean-Francois Aubry, Jason Sheehan

**Affiliations:** 1 University of Virginia, Charlottesville, Virginia, United States; 2 Focued Ultrasound Foundation, Charlottesville, Virginia, United States; 3 Institut Langevin, Paris, France

## Background/introduction

The purpose of this study was to investigate the thresholds for inducing two possible means of tissue destruction with low frequency Magnetic Resonance guided Focused Ultrasound (MRgFUS): either mechanical lesioning in presence of ultrasonic cavitation or pure thermal lesioning (without cavitation).

## Methods

Ten craniectomized pigs where sonicated with an ExAblate4000 Neuro (InSightec, Haifa, Israel) operated with a 220Hz array made of 1024 transducers. For each animal, a thermal lesion was aimed on the right thalamus, while a cavitation (mechanical) lesion was aimed on the contralateral side. For thermal lesioning, 40s duration sonications were performed and the acoustical energy ranged between 5600 J and 12000 J. For mechanical lesioning, 20s duration sonications were performed and similar total acoustic energy was used: energy ranged between 6000 J and 14000 J (power was increased to compensate for the short sonication time). Signals collected by two passive cavitation detectors (custom made by Insightec) were stored in memory during each sonication and cavitation activity was integrated within the bandwidth of the detectors, from 50 kHz to 182 kHz. 2D MR thermometry was performed during treatment. T1-weighted pre- and post-Gadolinium contrast-enhanced, T2-weighted, T2*-weighted, gradient echo and FLAIR were acquired after treatment. Pigs were euthanized immediately after the last series of MR imaging. Pig brains were harvested and fixed in formalin solution. Histology was performed to identify lesions.

## Results and conclusions

For thermal lesioning, the peak temperature at focus ranged between 49°C and 59°C. All thermal lesions were induced for peak temperature higher than 53°C. For mechanical lesioning, the peak temperature at focus ranged between 50°C and 57°C. Passive cavitation signals exhibited three main types of signal interpreted as follows: no cavitation, stable cavitation and inertial cavitation. Pure thermal lesions, as assessed by histology, could be generated with low frequency ultrasound. Such lesions showed up on T2 MR post-treatment images as a hypointense core surrounded by a hyperintense ring.

Mechanical lesions where associated with hemorrhages. The size of the hemorrhages measured on gross histology correlated with cavitation activity (R2=0.74) and a threshold for cavitation activity of 0.09 V.Hz (given the sensitivity of the Insightec cavitation detector or the frequency range) was found to divide the experiments into two separate groups: with and without hemorrhage. This work demonstrates that low frequency ultrasound can induce thermal lesions in the brain of living swines without hemorrhage. This work paves the way towards passive-cavitation-based automatic shutdown of low frequency ultrasound for safe ablation.

